# A case report of empyema caused by *Enterococcus gallinarum*

**DOI:** 10.1186/s12879-024-09531-6

**Published:** 2024-08-01

**Authors:** Min Liu, Jixiang Liu, Juanjuan Wu, Shuang Liu, Lu Sun, Fajiu Li, Chenghong Li

**Affiliations:** 1 Department of Pulmonary and Critical Care Medicine, Affiliated Hospital of Jianghan University, No.168 Hongkong Road, Wuhan, 430000 Hubei China; 2 National Center for Respiratory Medicine; State Key Laboratory of Respiratory Health and Multimorbidity; National Clinical Research Center for Respiratory Diseases; Institute of Respiratory Medicine, Chinese Academy of Medical Sciences; Department of Pulmonary and Critical Care Medicine, Center of Respiratory Medicine, China-Japan Friendship Hospital, No 2, East Yinghua Road, Beijing, 100029 China; 3 Institute of Pulmonary Vascular Diseases, Jianghan University, No.168 Hongkong Road, 430000 Wuhan, Hubei China

**Keywords:** *Enterococcus gallinarum*, Empyema, Immunodeficiency, Thoracoscopy

## Abstract

**Background:**

*Enterococcus gallinarum* is an infrequently intestinal symbiotic pathogen associated with nosocomial infection in immunocompromised individuals. To date, rare cases of pulmonary infection attributable to *Enterococcus gallinarum* were reported. Herein, we presented the first case of empyema resulting from *Enterococcus gallinarum* infection.

**Case presentation:**

An 81-year-old male presented with fever and dyspnea upon admission. Chest CT scan and thoracic ultrasonography confirmed the presence of right pleural effusion. Thoracoscopy revealed extensive adhesion, purulent fluid, and necrotic materials within the thoracic cavity. *Enterococcus gallinarum* was identified through pleural effusion culture. The patient underwent an intrathoracic injection of urokinase along with thoracic drainage. Following surgery, He took oral linezolid for over one month. Undergoing comprehensive treatment, the patient exhibited favorable recovery.

**Conclusions:**

We reported the first case of empyema due to *Enterococcus gallinarum* infection. It should be suspected in patients with impaired immune function and invasive therapies, without responding to conventional anti-infectious treatment.

## Background

As an opportunistic pathogen such as *Enterococcus gallinarum*, it causes nosocomial infection among immunosuppressed hosts. Recently, owing to the rise in broad-spectrum antibiotics and invasive procedures, *Enterococcus gallinarum* infection and multi-drug resistance have garnered increasing attention [1]. Previous case series reported extra-intestinal infections such as endocarditis [2], meningitis [3], peritonitis [4], and cholangitis [5] attributed to *Enterococcus gallinarum*. Currently, there is no report of respiratory system infection caused by *Enterococcus gallinarum*. Here, we presented a case of empyema due to *Enterococcus gallinarum* infection.

## Case presentation

An 81-year-old male was transferred to our hospital because of fever and dyspnea. In the past three years, this patient presented to the medical facility several times with recurrent choledocholithiasis complicated by cholangitis. One week prior to admission, he was readmitted for an acute attack and underwent percutaneous cholecystostomy with balloon dilation. Following surgery, the patient presented with fever. Despite receiving empirical anti-infectious therapy, the fever persisted and dyspnea ensued. The blood culture was found to be positive for a Gram-positive coccus. For further diagnosis and treatment, she was referred to our hospital. He had a history of arterial hypertension, coronary artery disease accompanied by atrial fibrillation, chronic obstructive pulmonary disease, and iron deficiency anemia. He had undergone cholecystectomy and Billroth II subtotal gastrectomy. Additionally, he had a 50-pack-year smoking history and had quit 18 years before the presentation.

On arrival, he presented with a temperature of 38.6℃, a blood pressure of 135/90 mmHg, a pulse rate of 90 beats per minute, a respiratory rate of 20 breaths per minute, and an oxygen saturation of 93% while breathing room air. Auscultation of the chest revealed absent breath sounds and dullness to percussion over the right lower lung field. During abdominal examination, tenderness was noted in the right upper quadrant without rebound pain. Additionally, bilateral pitting edema was present in the lower extremities.

**Fig. 1:**
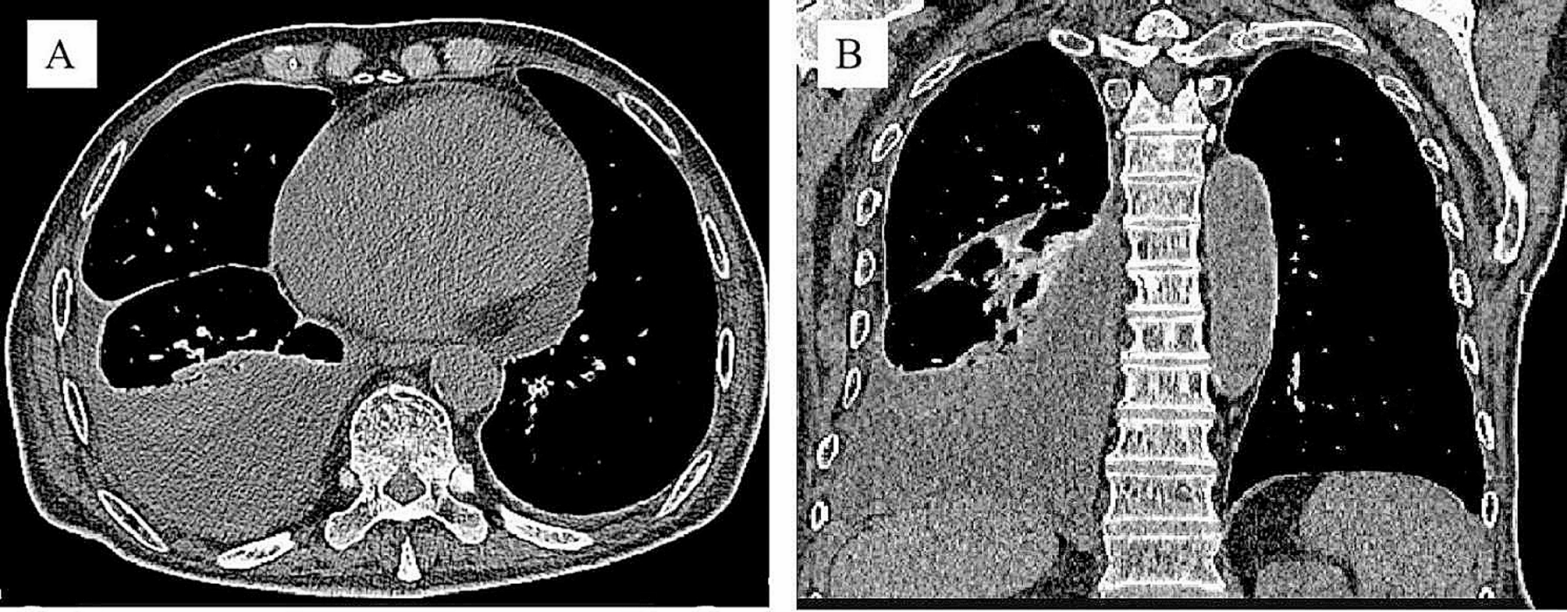
Radiological images of the chest CT. **A**, Axial scan showed a large right-sided pleural effusion. **B**, Coronal scan showed the pleural effusion was partially encapsulated

**Fig. 2:**
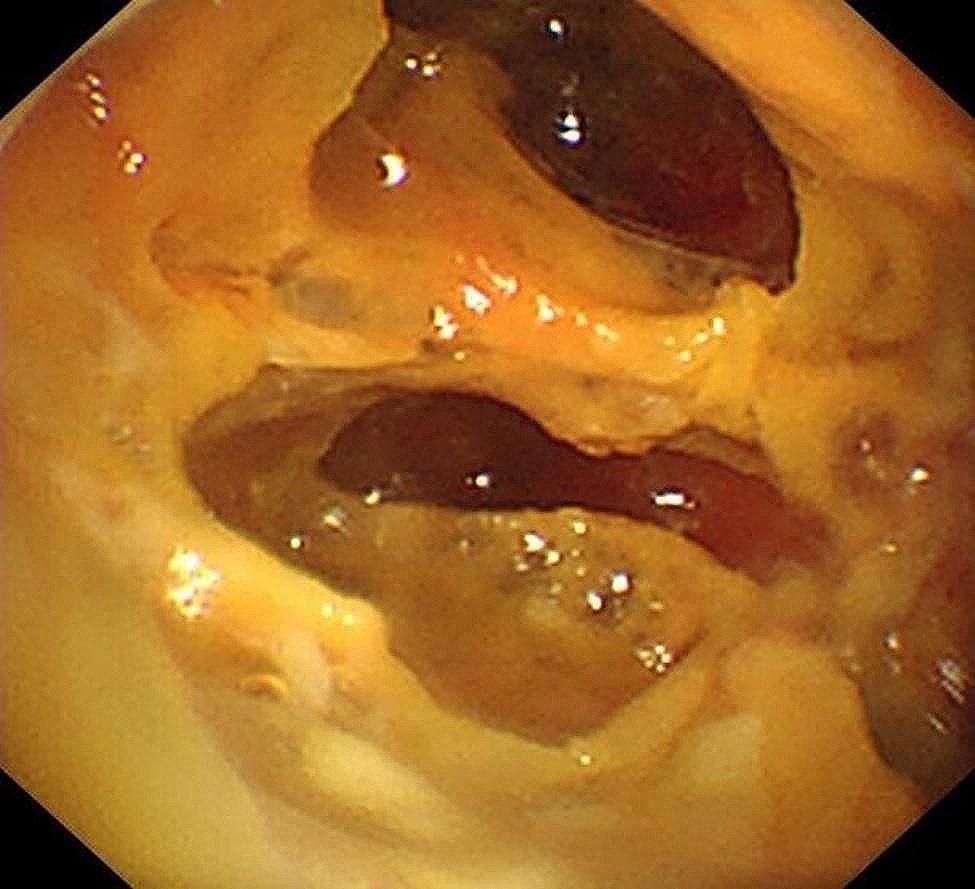
Medical thoracoscopy revealed the pleural thickening and adhesion, with extensive necrosis attaching to the surface of the parietal, visceral, and diaphragmatic pleura

**Fig. 3:**
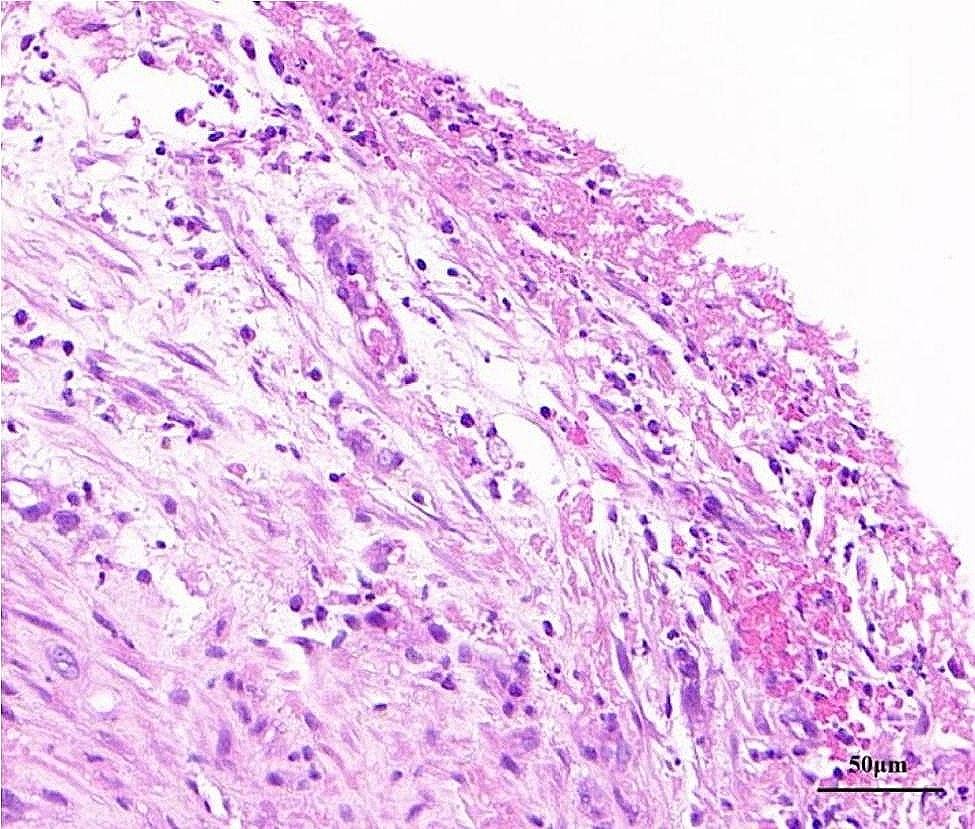
Hematoxylin and eosin staining revealed granulation with fibrinous exudation

Laboratory investigations showed white blood cell count 12.08 × 10^9^/L (normal value 4.0–10.0 × 10^9^/L) with neutrophils 11.6 × 10^9^/L. C-reactive protein significantly increased to 123.79 mg/L, accompanied by a mild elevation of procalcitonin to 0.26 ng/ml. Magnetic resonance cholangiopancreatography indicated mild dilation of the intrahepatic and extrahepatic bile ducts and multiple gallstones in the common bile duct. Chest CT scan showed a right subpulmonic effusion (Fig. 1). Thoracic ultrasonography confirmed an anechoic area with an anterior-posterior diameter of approximately 8.2 cm, exhibiting striped light bands and grid-like lesions within. Thoracoscopy showed extensive adhesion and purulent fluid, accompanied by necrotic materials within the thoracic cavity (Fig. 2). Analysis of pleural fluid revealed exudative pleural effusion, with a nucleated cell count of 6.2 × 10^9^/L, glucose level at 0.74 mmol/L, total protein concentration at 36.4 g/L, albumin concentration at 20.2 g/L, lactate dehydrogenase at 4032 U/L, and adenosine deaminase at 16.1 U/L. Cytology revealed the presence of inflammatory cells, including lymphocytes, neutrophils, and macrophages. *Enterococcus gallinarum* was identified in the pleural effusion culture, showing natural resistance to vancomycin but sensitivity to ampicillin, teicoplanin, penicillin G, ciprofloxacin, linezolid, and levofloxacin. Pleural biopsy showed granulation with fibrinous exudation (Fig. 3).

The patient underwent thoracic drainage and intrathoracic urokinase injection. Given the history of allergy to penicillin and sulfa, linezolid, as the most sensitive of the remaining antibiotics, was administered (600 mg twice daily) after the thoracic drainage. One week later, his temperature returned to normal and dyspnoea was significantly relieved. Oral linezolid was continued for one month after discharge. A follow-up after six months showed complete resolution of pleural effusion according to the chest CT scan.

## Discussion

*Enterococcus gallinarum* is a catalase-negative, facultative anaerobic, chain-arranged Gram-positive coccus isolated from the intestines of chickens [6]. As an opportunistic pathogen, *Enterococcus gallinarum* could colonize mesenteric veins, lymph nodes, liver, and spleen through an impaired intestinal barrier, leading to autoimmune diseases in susceptible individuals [7]. Additionally, *Enterococcus gallinarum* could also migrate to distant organs and cause systemic infections. It is the first case of empyema due to *Enterococcus gallinarum* infection which responded well to linezolid and had a favorable prognosis.

The gut barrier, acting as a selective gatekeeper, separates internal organs from trillions of intestinal microbes. Perturbations in host defenses and alterations in microbial community composition lead to pathological breaches. Once the intestinal barrier is compromised, increased intestinal permeability may facilitate the translocation of pathogenic microorganisms and their metabolites from the gut to other organs or the bloodstream, contributing to subsequent infection [8]. In addition, the evolution of the microbiota promotes immune evasion and bacterial translocation [9]. Patients undergoing invasive surgery or immunosuppression are also particularly susceptible. The patient in this report was elderly with multiple underlying diseases. He had received repeated courses of broad-spectrum antibiotics and had recently undergone an invasive procedure. These risk factors may predispose to gut barrier disruption, thereby facilitating the translocation of *Enterococcus gallinarum*.

*Enterococcus gallinarum*, a commensal of the human gastrointestinal tract, could occasionally cause severe infections especially in patients with bacteraemia and immunosuppressive hosts. It has been reported that the 30-day mortality rate of patients with bacteremia caused by Enterococcus gallinae was 29% [10]. Among the case reports of *Enterococcus gallinarum* meningitis, the purulent cerebrospinal fluid was reported in one patient with immune impairment [2]. The duration of the translocation and the microbial burden in the tissue may be important factors affecting the phenotype of disease. Symptoms associated with *Enterococcus gallinarum* infection might be nonspecific. The diagnosis of empyema attributed to *Enterococcus gallinarum* is confirmed through the culture of pleural effusion. Next-generation sequencing of pleural fluid could be performed to improve the sensitivity of diagnosis if necessary. Thoracoscopy may be beneficial to rule out differential diagnosis such as malignant tumor or tuberculous pleurisy.

The Enterococcus genome is characterized by a robust antimicrobial resistance pattern, with vancomycin resistance being an inherent feature. *Enterococcus gallinarum* specifically harbors the vanC resistance gene, demonstrating a high level of vancomycin resistance. Recent isolations of strains carrying vanA and vanB genes, in addition to vanC, have shown elevated resistance to both vancomycin and teicoplanin [1, 11]. Moreover, *Enterococcus gallinarum* strains have occasionally exhibited resistance to linezolid, with recent identifications of linezolid-resistant strains possessing the cfr, optrA, and poxtA genes [12]. Sometimes, it may still be ineffective, even if it is sensitive in vitro. This patient has received an appropriate dose of linezolid and achieved satisfactory improvement after four weeks of treatment. Suboptimal treatment may result in pleural scarring due to visceral pleural fibrosis and lung function deficits or even death. Failure of treatment often requires surgery, which further increase the cost and the burden of the disease. This case highlights the need for healthcare professionals worldwide to raise awareness of empyema caused by rare pathogens such as *Enterococcus gallinarum*.

In conclusion, empyema resulting from *Enterococcus gallinarum* is rare, and it should be suspected especially in immunosuppressive hosts. Timely detection and accurate diagnosis, as well as rational use of antibiotics, are critical for the treatment of *Enterococcus gallinarum* infection.

## Data Availability

All the data of this case is contained within the manuscript.
